# Learning impacts on elementary education students during physical and social distancing due COVID-19

**DOI:** 10.1590/2317-1782/20212020373

**Published:** 2022-06-27

**Authors:** Alexandre Lucas de Araújo Barbosa, Ana Beatriz Leite dos Anjos, Cíntia Alves Salgado Azoni

**Affiliations:** 1 Programa de Pós-graduação em Psicologia, Universidade Federal do Rio Grande do Norte – UFRN - Natal (RN), Brasil.; 2 Programa de Pós-graduação em Fonoaudiologia, Universidade Federal do Rio Grande do Norte – UFRN - Natal (RN), Brasil.

**Keywords:** COVID-19, Learning, Neurodevelopmental Disorders, Reading, Students

## Abstract

The social and physical isolation caused by COVID-19 changed the world’s educational reality. This article aimed to identify publications in the world literature that report the impacts of such isolation on the learning process of children and adolescents in elementary education. The results showed that among the fourteen studies analyzed, there is an alert to the students in situations of social vulnerability, with the worse repercussions on girls at risk for early pregnancy and overload of domestic work, as well as academic losses due to the absence of food in the school context of those who depends on the school to survive. There are still few studies that give direction to students with educational special needs and, in Brazil, there are no studies correlating the learning process with elementary education students during the COVID-19 pandemic.

## INTRODUCTION

Learning is a process that allows the appropriation of knowledge from social world experiences^(1)^. Learning in the school environment is important for a child’s development due to cognitive and linguistic elements for formal teaching of written language, social relations to which the child is exposed, and many other necessary elements in daily education context. The novel coronavirus, agent of COVID-19, which causes impairments such as fever, cough, fatigue, headache, breathing difficulties and, in more severe cases, Severe Acute Respiratory Syndrome (SARS), has reflected on the closing of schools for promoting physical distancing of people. Due to its nature of transmission through respiratory secretions, social isolation became a way to fight the disease in places with community transmission of the virus due to the absence of medications proven efficient^(2)^.

Until June 2021, there were over 173 million confirmed cases and three million deaths worldwide, with an exponential daily growth. In Brazil, during the same period, there were over 16 million cases and more than two hundred thousand deaths^(3)^. Therefore, as prevention measure to the virus dissemination in crowded conditions, school classes, offices, large events, and public markets, for example, were suspended^(4)^.

Around 60% of schools canceled in person classes, jeopardizing over a billion students throughout the world and around fifty-two million in Brazil, according to data by UNESCO^(5)^. Seeking to provide continuity to teaching-learning process, several schools have adopted digital resources; however, their effect is limited and requires joint efforts between teachers and family members. The limitations of online teaching include difficulties in teaching of skills, hampered receiving feedback by students, limited attention span, and absence of discipline in following up the classes. In addition, special attention should be given to inequalities existing in systematic education since students of low socioeconomic levels face difficulties in accessing the technological resources required to follow up activities and are unable to be stimulated during the isolation period^(6)^.

The use of internet and social networks in the school environment and teaching-learning relation had been a reality since before the pandemic. Although the internet is present, a better exploitation of content requires the help and guidance of an educator. A study conducted in 2010 gathered data about adolescents from schools of both public and private networks, where 61% of the students at public schools and 63% at private schools accessed the internet every day. Such figures reveal the absence of disparity in digital access among students of different social classes. However, regarding the use of networks for searches in the context of school activities, 93% of the students of the private network used the internet, in contrast with 65% of the public school students. Geography, history, and Portuguese, respectively, were the disciplines with more frequently found material available^(7)^.

The literature shows that a considerable time without stimulation causes negative impacts on children’s learning. An example of such impact is the phenomenon known as Summer Learning Loss (SLL), defined as a loss in school skills over the period of school vacations for both reading and remaining skills, like mathematics. According to researchers, the effect can be larger for low-income children, especially due to the absence of resources and difficulty in accessing materials outside the school environment^(8)^.

A study published by Menard & Wilson (2014) concluded that the SLL also occurs in children with learning disorder resulting impaired reading. By comparing a group of 30 children with the mentioned diagnosis and another of 30 children with typical development, the authors found that the group with the learning disorder presented greater losses in decoding, fluency, and reading speed skills. The damages were also observed in the group of typical children, however at lower magnitude^(9)^.

Another example of the damages caused by a period without stimulation on children’s learning is the higher absence rate. A study with 5,103 children at elementary school and 4,983 at pre-school has identified more difficulties in reading, writing, and mathematics by those who did not attend school regularly due to adverse situations, like problems with family members or health. Thus, the pandemic period hampers or precludes school attendance, and more severe long-term consequences should be expected, such as lower learning retention during the school year or even school dropout^(10)^.

Therefore, evidence on the relation between absence of stimulus and learning is clear since the apprehended skills tend to show damages rapidly upon the absence of practice. Such consequence is especially visible in students with lower level of previous proficiency at the skill in question^(11)^.

Such data reveal that a period without school stimulation has negative impacts on academic learning. Thus, this study has been guided by the following research question: What are the consequences of social physical isolation during COVID-19 pandemic on pre-school and school learning?

## PURPOSE

Our goal is to identify literature research reporting the impacts of isolation on learning of children and adolescents at elementary education.

## RESEARCH STRATEGIES

This research is a scope review aimed at finding new evidence and productions on a recent topic^(12)^, in this case, the impacts of social physical isolation and suspension of school classes on pre-school and school learning.

This research was supported by the databases of PubMed, Education Resources Information Center (ERIC), *Literatura Latino-Americana e do Caribe em Ciência das Saúde* (LILACS), ScienceDirect, PsycINFO, and Scopus. Keywords were selected through Medical Subject Headings (MeSH), Thesaurus, Descriptors in Health Sciences (DeCS), and Open Grey (grey literature) using Boolean operators AND and OR combined with the following descriptors. We traced multiple searches to generate a larger number of papers on the topic due to the recentness of the subject: “COVID-19 OR pandemics” AND “learning OR education”; “COVID-19 OR pandemics” AND “child OR children”; “COVID-19 OR pandemics” AND “preschool OR preschool children”; “COVID-19 OR pandemics” AND “learning OR education”; “COVID-19” AND “students OR child OR pre-school”. The Rayyan QCRI tool was applied to manage the references and selection of papers with blinding of the authors.

## SELECTION CRITERIA

We included papers published from December 1, 2019, to July 15, 2020 (duration of pandemic until the beginning of the research), in English, Portuguese, and Spanish and covering all designs. All studies should present some of the following characteristics: papers with sample of pre-school or elementary education school, with or without neurodevelopment alterations, in the context of social physical isolation due to the COVID-19 pandemic; research indicating recommendations or consequences of social isolation/COVID-19 on school learning; papers reporting adaptations for the continuity of students’ learning over the mentioned period; studies describing school performance of individuals subjected to social isolation/COVID-19. We excluded papers whose topic addressed the learning of students of higher education during the pandemic period or description focused on teaching management process for remote classes, in addition to those that directly approached mental health in school, considering that other variables could be associated.

After the search, the papers were blindingly analyzed by the authors according to title and abstract. All articles were fully read. The inclusion or exclusion was discussed among authors in two meetings.

## DATA ANALYSIS

All studies selected for the final sample were analyzed considering the study population and related learning impacts as consequence of the COVID-19 pandemic.

## RESULTS

**Figure 1:**
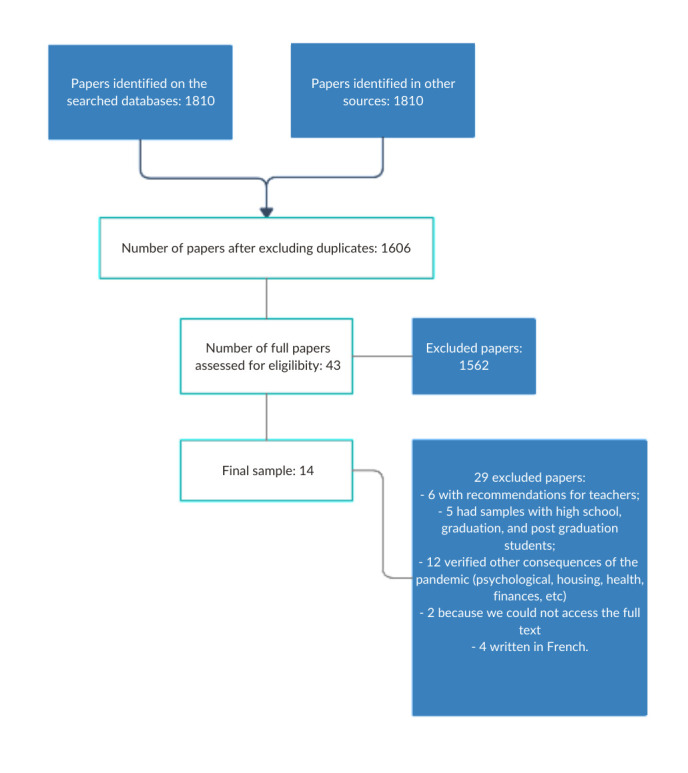
Flowchart of papers analysis

Initial search resulted in 1810 references, out of which 1606 remained after excluding repeated titles. Screening by title and abstract allowed to limit the number to 43 papers. Among these, according to the selection criteria, 14 composed the final sample. Flowchart (Figure 1) illustrates the stages.

**Figure 2:**
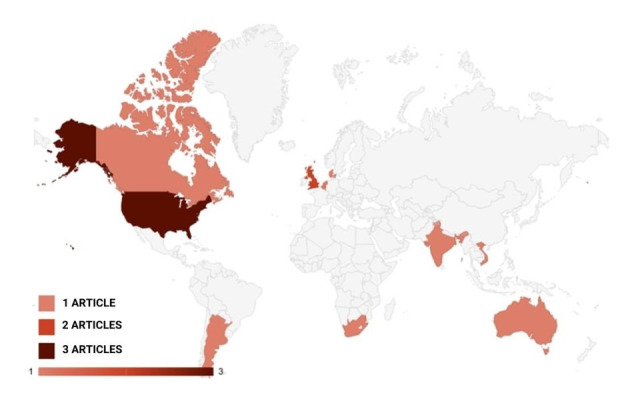
Map of distribution by country and publication of the papers selected

The final papers were fully read and analyzed according to our objectives. Tables 1 and 2 summarize our findings and Figure 2 illustrates the distribution of the publications by country.

**Table 1:** Impacts on learning, according to the papers analyzed

**Reference**	**Population**	**Impact on learning**
Frenette et al.^(13)^	Individuals below 18 years of age	Internet access difficulties; small number of devices available; learning gaps; reduced class time
Young and Donovan^(14)^	Students with special education needs	Students with severe disorders may face difficulties regarding their needs; severe difficulties derived from family environment; self-regulation difficulties during class; greater use of assistance technologies
Russell^(15)^	Students from children’s education to undergraduate	Disadvantages for students of low socioeconomic level and of rural areas; learning gaps; anxiety at the new teaching-learning configuration
Sevilla Vallejo e Ceballos Marón^(16)^	Students at elementary education	Avoiding school activities; difficulties in understanding activities; reading difficulties (vocabulary and text comprehension)
Jæger and Blaabaek^(17)^	Students between 0 and 16 years of age	Families of low socioeconomic level tend to borrow fewer books from the library
Fitzgerald et al.^(18)^	Students at elementary school I	Use of remote teaching; probable damages regarding literacy and numeracy
The WHO-UNICEF-Lancet Commissioners^(19)^	Vulnerable students	Learning gaps; school dropout due to children’s pregnancy indices
Burzynska and Contreras^(20)^	Female students	School dropout due to early pregnancy, sexual exploitation, and forced marriage; less time dedicated to studies due to domestic labor
Armitage and Nellums^(21)^	Students of teaching children’s	Severe inequalities of access to education; school dropout due to children’s labor, violence against children, and early pregnancy
Khattab et al.^(22)^	Students at children’s education	Damage to “at risk” children; inequalities of access to internet and technological devices;
Van Lancker and Parolin^(23)^	Socioeconomic vulnerable students	Food insecurity leading to academic damages; inequalities of access between low and high-income classes; inadequate domestic environment for classes
Spaull^(24)^	South-African students	Difficulties of access to technology and education materials
Tran et al.^(25)^	Students at elementary education	Students at school public dedicate less time to study during the pandemic
Khattar et al.^(26)^	Indian students	Difficulty to adapting to online education

**Table 2:** Categorization of learning impacts.

**Type of impact**	**Studies**
Difficulties in accessing technological resources	Frenette et al.^(13)^; Fitzgerald et al.^(18)^; Khattab et al.^(22)^; Spaull^(24)^
Gender inequality	The WHO-UNICEF-Lancet Commissioners^(19)^; Burzynska and Contreras^(20)^; Armitage and Nellums^(21)^
Students with special education needs (SEN)	Young and Donovan^(14)^
Socioeconomic issues	Russell^(15)^; Jæger e Blaabaek^(17)^; Armitage and Nellums^(21)^; Van Lancker and Parolin^(23)^
Difficulties in adapting to online education	Khattar et al.^(26)^; Tran et al.^(25)^; Sevilla Vallejo e Ceballos Marón^(16)^

## DISCUSSION

Closing of schools resulted in a considerable level of protection for both teachers and students, but required remote teaching as a fast and temporary alternative. The impact of inconsistencies and absence of standardization of such teaching model is yet to be revealed on long term^(18)^.

All studies included for analysis frequently mentioned the difference of impacts of the suspension of classes on high- and low-income children. The learning gap derived from the period in question is inevitable; however, for children of high social classes, the effect seems to be softer since those face fewer difficulties in continuing their education process through digital routes^(19)^. Conversely, low-income children do not have a proper environment to attend classes or do the activities proposed by the teacher^(23)^.

For low-income children, the access to tools required for digital education is very limited. First, there is a difficulty in acquiring device connectable to the internet. Thus, the number of devices available per family is reduced, which also affects the period of time provided for the child to attend the classes since the device has to be shared^(13,24)^.

Many low-income and rural families may present limitations related to internet connection, thus hampering communication with teachers for receiving class instructions, often sent via email^(15)^. In Brazil, only half of the households has access to internet in the rural zone, while in the urban zone, the number increases by 83.8%. A survey by the Brazilian Institute of Geography and Statistics (2019) pointed to the following main reasons for not using the internet: expensive internet access service; none of the inhabitants knew how to use the internet or the internet access service was not available in the area^(27)^. Such scenario is vastly different in countries like Canada, where only 1.2% of the population below 18 years old have no connection in the house^(13)^.

Another difference between these two strata of population is that the families of higher socioeconomic classes tend to borrow more books from the library, both digital versions and hard copies. Such scenario has intensified during the pandemic causing an increase in the inequality of access to education resources outside school^(17)^.

In addition to accessing education resources, many children who attended school depended on it for healthy food, since food at school is one of the main nutrition sources for children at poverty situation. The suspension of classes has interrupted these children’s access to quality food, which may have significantly affected their cognitive development, especially for the younger ones^(19)^, since the meals offered by the school are positively associated with good academic performance^(23)^.

A study conducted with students at basic education investigated the relation between free food offered at schools and academic performance. For this purpose, the authors established a longitudinal analysis of the results of exams measuring language and mathematics skills. They found that the offer of free or low-price food during the school period results in better performance in the exams, both for students at poverty situation and for those economically stable^(28)^. Such data allow to interpret that during the pandemic, students who depended upon food offered at school were at risk regarding the development of academic skills, in addition to nutritional and survival matters.

Part of the papers also focused on the effect of gender and related consequences on learning. Girls presented larger risk of not returning to school due to higher rate of early pregnancy, forced housing, and sexual exploitation. The authors reinforce that during the Ebola epidemic, the number of pregnant girls retuning to classes was remarkable, where many of them had been prohibited to return to school. Another important element is that during the pandemic, girls spend at least 40% more time on domestic errands than boys, which reduces the time dedicated to studies and appreciation of education by parents^(20)^.

The transition of on-site classes to online classes has also resulted in less training time and impaired the participation of some students in the activities proposed by teachers. On long term, class time restriction may cause damages to certain reading, mathematics, and sciences performance skills^(13)^. Finally, an element explored in only one paper refers to the effects on students with special education needs, since those benefit significantly from socialization provided by on-site classes^(22)^ and from the assistance offered by auxiliary teachers and daily adaptations, which remains a challenge to teachers in the virtual context.

Despite the contributions of the published papers to enlarge the knowledge of damages on children’s learning, we found no studies addressing specific elements of school learning for reading, writing, and mathematics. It is possible that after the end of the pandemic, further studies will be conducted due to the children’s return to classroom, which will allow to observe long-term impacts of the suspension on the learning process.

None of the published studies was performed in Brazil, which highlights the need to conduct research supported by a perspective of education elements due to the novel coronavirus pandemic considering the continental size of the country and its severe social inequality.

## CONCLUSION

This review allows to learn that the suspension of classes due to the novel coronavirus COVID-19 pandemic will have consequences on learning of children with or without neurodevelopment disorders. We neither found studies describing learning on the perspective of academic performance of reading, writing, and mathematics, nor any research carried out in Brazil. The focus of the studies was the main effects that will reflect on learning due to differences of access between high- and low-income children based on inequal conditions of teaching-learning and technological resources and education materials for following up remote classes and study at home. Other consequences that influence learning reported as essential elements in students’ life refer to absence of healthy food, which hampers children’s cognitive functioning; early pregnancy, jeopardizing continuous attendance to classes; sexual exploitation of girls, children’s labor, and less class time, all aggravating the influence of emotional issues on learning.
