# Supplementary material for: Learning impacts on elementary education students during physical and social distancing due COVID-19
Source: Codas. 2022 Jun 27;34(4):e20200373. doi: 10.1590/2317-1782/20212020373 (PMC9886128; doi:10.1590/2317-1782/20212020373)
Supplement: Translation

# Translation: Impactos na aprendizagem de estudantes da educação básica durante o isolamento físico social pela pandemia do COVID-19

**Authors:** Alexandre Lucas de Araújo Barbosa, Ana Beatriz Leite dos Anjos, Cíntia Alves Salgado Azoni

**Affiliations:** 1 Programa de Pós-graduação em Psicologia, Universidade Federal do Rio Grande do Norte – UFRN - Natal (RN), Brasil.; 2 Programa de Pós-graduação em Fonoaudiologia, Universidade Federal do Rio Grande do Norte – UFRN - Natal (RN), Brasil.

**Keywords:** COVID-19, Aprendizagem, Transtornos do Neurodesenvolvimento, Leitura, Estudantes

## Abstract

O isolamento físico social ocasionado pela pandemia do COVID-19 trouxe mudanças na realidade educacional mundial. O objetivo deste artigo foi identificar na literatura mundial artigos que relatam os impactos deste isolamento na aprendizagem de crianças e adolescentes da educação básica. Os resultados evidenciaram que, dentre os quatorze estudos analisados, há um alerta para os estudantes em situação de vulnerabilidade social, com pior repercussão em meninas em situação de risco para gestação precoce e sobrecarga de trabalho doméstico, assim como prejuízos acadêmicos em virtude da ausência da alimentação no contexto escolar daqueles que dependem da escola para sobrevivência. Ainda há poucos estudos que direcionam a atenção para estudantes com necessidades educacionais especiais e, no Brasil, não há estudos relacionados à aprendizagem com estudantes da educação básica durante a pandemia da COVID-19.

## INTRODUÇÃO

A aprendizagem é um processo que possibilita a apropriação de conhecimentos, a partir de experiências do mundo social^(1)^. A aprendizagem no ambiente escolar é de importância para o desenvolvimento da criança, visto os aspectos cognitivos e linguísticos para o ensino formal da linguagem escrita, aspectos das relações sociais a que está exposta e tantos outros elementos necessários no contexto educacional diário. O novo coronavírus, causador da COVID-19, que traz comprometimentos como febre, tosse, fadiga, dores de cabeça, dificuldades respiratórias e, em casos mais graves, a Síndrome Respiratória Aguda Grave (SARS), repercutiu no fechamento das escolas em função do distanciamento físico de pessoas. Em função disso, por se tratar de uma transmissão por secreções respiratórias, o isolamento social tem sido uma das formas de combate à doença em locais em que há transmissão comunitária do vírus, em razão da ausência de medicamentos comprovadamente eficazes para combatê-la^(2)^.

Até junho de 2021, são mais de 173 milhões de casos confirmados e três milhões de mortes ao redor do globo, com crescimento diário exponencial. No Brasil, nesse mesmo período, foram mais de 16 milhões de casos e mais de duzentas mil mortes^(3)^. Assim, como medida preventiva à disseminação em situações de aglomeração, foram suspensas aulas escolares, funcionamento de escritórios, eventos de grande porte e mercados públicos, por exemplo^(4)^.

O cancelamento das aulas ocorreu em cerca de 60% das escolas, prejudicando mais de um bilhão de estudantes ao redor do mundo e cerca de cinquenta e dois milhões no Brasil, de acordo com dados da UNESCO^(5)^. Como forma de dar continuidade ao processo de ensino-aprendizagem, diversas escolas têm adotado recursos digitais, porém seu efeito é limitado e requer a união de esforços entre os professores e os familiares. Algumas limitações do ensino online são: dificuldades para ensinagem de habilidades, dificuldades de receber *feedback* dos estudantes, tempo de atenção limitado e falta de disciplina no acompanhamento das aulas. Além disso, é necessária atenção especial às desigualdades existentes no sistema educacional, visto que estudantes de baixo nível socioeconômico terão dificuldades de acesso aos recursos tecnológicos necessários para acompanhar as atividades, impossibilitados de receber estimulação durante este período^(6)^.

O uso da internet e redes sociais no ambiente escolar e na relação ensino-aprendizagem é uma realidade desde antes da pandemia. Apesar da internet estar presente, para um melhor aproveitamento do conteúdo, é necessário auxílio e orientação de um educador. Um estudo realizado em 2010 reuniu dados de adolescentes de escolas da rede pública e privada, nos quais 61% dos alunos de escola pública e 63% de escola privada acessam a internet todos os dias. Tal fato revela que não houve disparidade no acesso digital entre alunos de diferentes classes sociais. No entanto, ao se referir ao uso das redes para realização de pesquisas no desenvolvimento das atividades escolares, 93% dos alunos da rede privada utilizam a internet, enquanto 65% daqueles de escola pública também o fazem. As disciplinas que os alunos mais encontram material disponível são geografia, história e português, respectivamente^(7)^.

Existem evidências na literatura indicando que um período de tempo considerável sem estimulação causa impactos negativos na aprendizagem infantil. Um exemplo deste impacto é o fenômeno conhecido como *Summer Learning Loss (SLL)*, definido como uma perda nas habilidades escolares durante o período de férias acadêmicas, podendo ser observado tanto nas habilidades de leitura, quanto nas demais como a matemática. De acordo com os pesquisadores, o efeito pode ser maior para crianças de baixa renda, principalmente pela falta de recursos e dificuldade de acesso aos materiais quando longe do ambiente escolar^(8)^.

Um estudo publicado por Menard & Wilson (2014) concluiu que o *SLL* ocorre também em crianças com transtorno de aprendizagem com prejuízo na leitura. Ao comparar um grupo de 30 crianças com o diagnóstico, com outro de 30 crianças com desenvolvimento típico, os autores constataram que o grupo com transtorno de aprendizagem apresentou maiores perdas nas habilidades de decodificação, fluência e velocidade de leitura. Os prejuízos foram igualmente observados no grupo de crianças típicas, mas em menor magnitude^(9)^.

Outro exemplo de como o período ausente de estimulação pode prejudicar a aprendizagem infantil é o número elevado de faltas. Em um estudo com 5.103 crianças do ensino fundamental e 4.983 pré-escolares, identificou mais dificuldades na leitura, escrita e matemática naquelas que não frequentavam regularmente a escola, devido às situações adversas como problemas familiares e de saúde. Assim, neste período de pandemia que dificulta ou impossibilita a assiduidade dos escolares, é possível esperar consequências maiores a longo prazo, como menor retenção da aprendizagem durante o ano letivo ou abandono escolar^(10)^.

Desta forma, são claras as evidências da relação entre ausência de estímulo e a aprendizagem, pois as habilidades aprendidas tendem a mostrar prejuízos rapidamente quando na ausência da prática. Esta consequência será especialmente notável em estudantes com menor nível de proficiência prévio na habilidade em questão^(11)^.

De acordo com os dados apresentados acima, é possível que um período ausente de estimulação escolar traga impactos negativos à aprendizagem acadêmica. Assim, esta pesquisa foi guiada pela seguinte pergunta de pesquisa: quais as consequências na aprendizagem de pré-escolares e escolares em decorrência do isolamento físico social durante a pandemia do COVID-19?

## OBJETIVO

O estudo em questão tem como objetivo identificar na literatura mundial artigos que relatam os impactos deste isolamento na aprendizagem de crianças e adolescentes da educação básica.

## ESTRATÉGIAS DE PESQUISA

Trata-se de uma revisão de escopo que tem o objetivo de observar novas evidências e produções acerca de um tema recente^(12)^. Neste caso, os impactos do isolamento físico social e suspensão das aulas escolares na aprendizagem de pré-escolares e escolares.

Esta pesquisa foi realizada nas bases de dados PubMed, *Education Resources Information Center* (ERIC), Literatura Latino-Americana e do Caribe em Ciências da Saúde (LILACS), ScienceDirect, PsycINFO e Scopus. As palavras-chave foram selecionadas por meio do *Medical Subject Headings* (MeSH), Thesaurus, Descritores em Ciências da Saúde (DeCS) e Open Grey (literatura cinzenta), utilizando os operadores booleanos AND e OR em combinação com os seguintes descritores. Foram traçadas múltiplas buscas, a fim de obter o maior número de artigos acerca do assunto, visto ainda ser um tema recente: “COVID-19 OR pandemics” AND “learning OR education”; “COVID-19 OR pandemics” AND “child OR children”; “COVID-19 OR pandemics” AND “preschool OR preschool children”; “COVID-19 OR pandemias” AND “aprendizagem OR educação”; “COVID-19” AND “estudantes OR criança OR pré-escolar”. Para a administração das referências e seleção dos artigos com cegamento entre os autores, foi utilizada a ferramenta *Rayyan QCRI*.

## CRITÉRIOS DE SELEÇÃO

Foram incluídos artigos publicados no período de 1 de dezembro de 2019 a 15 de julho de 2020 (período de duração da pandemia, até o início deste estudo), nos idiomas inglês, português e espanhol, de todos os delineamentos. Os estudos precisavam apresentar alguma das seguintes características: artigos com amostra de pré-escolares ou escolares da educação básica, com ou sem alterações do neurodesenvolvimento, em isolamento físico social devido a pandemia do COVID-19; estudos que indicaram recomendações ou consequências do isolamento social/COVID-19 na aprendizagem escolar; artigos que relataram adaptações realizadas para a continuidade da aprendizagem dos alunos durante o período citado; estudos que descreveram o desempenho escolar dos indivíduos submetidos ao isolamento social/COVID-19. Foram excluídos artigos nos quais o tema fosse a respeito da aprendizagem de estudantes do ensino superior no período de pandemia ou aqueles que a descrição se voltasse ao processo de manejo do ensino do professor nas aulas remotas; bem como aqueles que abordassem diretamente a saúde mental de escolares, considerando que outras variáveis podem estar associadas.

Após a busca, os artigos foram analisados de forma cega pelos autores, de acordo com seu título e resumo individualmente pelos autores. Em seguida, foi realizada a leitura completa dos textos. Devido a incongruência de opinião entre os artigos selecionados entre os autores, tanto na fase de análise de títulos e resumos, quanto na fase de artigos completos, foi necessária a realização de duas reuniões para discussão dos artigos e decisão final sobre a inclusão ou exclusão na pesquisa.

## ANÁLISE DOS DADOS

Todos os estudos selecionados para a amostra final foram analisados levando em conta a população de estudo e as consequências na aprendizagem descritas em razão da pandemia do COVID-19.

## RESULTADOS

A busca inicial resultou em 1810 referências, das quais restaram 1606 após a exclusão dos títulos repetidos. O rastreio pelo título e resumo possibilitou limitar esse número a 43 artigos. Destes, seguindo os critérios de seleção, 14 compuseram a amostra final. As etapas estão ilustradas no Fluxograma (Figura 1).

**Figura 1:** Fluxograma de análise dos artigos

Os artigos finais foram lidos na íntegra e analisados de acordo com os objetivos deste estudo. A Tabela 1 e a Tabela 2 resumem os achados e a Figura 2 demonstra a distribuição das publicações por países:

**Tabela 1:** Consequências na aprendizagem, de acordo com os artigos analisados

**Referência**	**População**	**Consequências na aprendizagem**
Frenette et al.^(13)^	Indivíduos abaixo dos 18 anos de idade	Dificuldades de acesso à internet; número de dispositivos disponíveis reduzido; *gaps* de aprendizagem; tempo de aula reduzido
Young e Donovan^(14)^	Estudantes com necessidades educacionais especiais	Estudantes com transtornos mais graves podem ter dificuldades quanto ao atendimento de suas necessidades; agravamento de dificuldades devido ao ambiente familiar; dificuldades em auto-regulação durante a aula; maior uso de tecnologias assistenciais
Russell^(15)^	Estudantes do ensino infantil à graduação	Desvantagens de estudantes de baixo nível socioeconômico e de áreas rurais; *gaps* na aprendizagem; ansiedade frente ao novo formato de ensino-aprendizagem
Sevilla Vallejo e Ceballos Marón^(16)^	Estudantes do ensino primário	Evitar a realização de atividades escolares; dificuldades de compreender as atividades; dificuldades de leitura (vocabulário e compreensão do texto)
Jæger e Blaabaek^(17)^	Estudantes de 0 a 16 anos	Famílias de baixo nível socioeconômico tendem a pegar menos livros na biblioteca
Fitzgerald et al.^(18)^	Estudantes do ensino fundamental I	Uso do ensino remoto; prováveis prejuízos quanto à literacia e numeracia
The WHO-UNICEF-Lancet Commissioners^(19)^	Estudantes em situações de vulnerabilidade	*Gaps* de aprendizagem; abandono escolar em decorrência dos índices de gravidez infantil
Burzynska e Contreras^(20)^	Estudantes do sexo feminino	Abandono escolar em decorrência de gravidez precoce, exploração sexual e casamento forçado; menor tempo dedicado aos estudos devido a trabalhos domésticos
Armitage e Nellums^(21)^	Estudantes do ensino infantil	Agravamento das desigualdades de acesso educacional; abandono escolar devido a trabalho infantil, violência contra a criança e gravidez precoce
Khattab et al.^(22)^	Estudantes do ensino infantil	Prejuízo para as crianças “em risco”; desigualdades de acesso a internet e dispositivos tecnológicos;
Van Lancker e Parolin^(23)^	Estudantes em situação de vulnerabilidade socioeconômica	Insegurança alimentar, o que leva a prejuízos acadêmicos; desigualdades de acesso entre baixa e alta classe socioeconômica; ambiente doméstico não adequado para atender às aulas
Spaull^(24)^	Estudantes sul-africanos	Dificuldades de acesso à tecnologia e materiais educacionais
Tran et al.^(25)^	Estudantes do ensino fundamental	Estudantes de escola pública dedicam menos tempo ao estudo durante a pandemia
Khattar et al.^(26)^	Estudantes indianos	Dificuldade de se adaptar ao ensino online

**Tabela 2:** Categorização das consequências na aprendizagem

**Tipo de consequência**	**Estudos**
Dificuldades de acesso a recursos tecnológicos	Frenette et al.^(13)^; Fitzgerald et al.^(18)^; Khattab et al.^(22)^; Spaull^(24)^
Desigualdade de gênero	The WHO-UNICEF-Lancet Commissioners^(19)^; Burzynska e Contreras^(20)^; Armitage e Nellums^(21)^
Estudantes com necessidades educacionais especiais (NEE)	Young e Donovan^(14)^
Questões socioeconômicas	Russell^(15)^; Jæger e Blaabaek^(17)^; Armitage e Nellums^(21)^; Van Lancker e Parolin^(23)^
Dificuldades de adaptação ao ensino online	Khattar et al.^(26)^; Tran et al.^(25)^; Sevilla Vallejo e Ceballos Marón^(16)^

**Figura 2:** Mapa de distribuição por países, de publicação, dos artigos encontrados

## DISCUSSÃO

O fechamento das escolas, por resultar em uma proteção considerável para professores e alunos, trouxe a necessidade de desenvolver uma rápida e temporária alternativa: o ensino remoto. Apesar disso, as inconsistências e ausência de padronização desse ensino só terá seu impacto revelado a longo prazo^(18)^.

Os estudos incluídos para análise fizeram frequente menção sobre a diferença do impacto na suspensão das aulas entre crianças de alta e de baixa renda. A lacuna na aprendizagem neste período é inevitável, porém para crianças de altas classes sociais o efeito parece ser menor, pois há menos dificuldades para a continuidade do processo educacional por vias digitais^(19)^. Por outro lado, crianças de baixa renda não possuem, em geral, ambiente adequado para assistir às aulas ou fazer as atividades prescritas pelo professor^(23)^.

Para crianças de baixa renda, o acesso às ferramentas necessárias para a educação digital é escasso. Primeiramente, há dificuldade quanto à aquisição de dispositivos conectáveis à internet. Desta forma, o número de dispositivos disponíveis por família é reduzido, o que afeta também o tempo em que a criança dispõe para as aulas, visto que é necessário dividir o uso do aparelho^(13,24)^.

Muitas famílias de baixa renda ou da zona rural podem ter limitações relacionadas à conexão com a internet, impossibilitando assim a comunicação com os professores para receber as instruções das aulas, frequentemente enviadas via email^(15)^. No Brasil, apenas metade dos domicílios utilizam a internet na zona rural, enquanto na zona urbana o número sobe para 83,8%. Em levantamento do Instituto Brasileiro de Geografia e Estatística (2019), os principais motivos para a não utilização da internet foram: o serviço de acesso à internet era caro, nenhum morador sabia usar a internet e o serviço de acesso à internet não estava disponível na área do domicílio^(27)^. Este cenário é diferente em países como o Canadá, no qual apenas 1,2% da população abaixo dos 18 anos não tem conexão em casa^(13)^.

Outra diferença entre estes dois estratos da população é o fato de que famílias de maiores classes socioeconômicas tendem a pegar mais livros emprestados na biblioteca, tanto nas versões digitais, quanto nas versões impressas. Durante a pandemia, isto se intensificou, causando um aumento na desigualdade de acesso a recursos educacionais fora da escola^(17)^.

Além do acesso a recursos educacionais, muitas crianças que frequentavam a escola dependiam dela para alimentar-se de forma saudável, sendo a alimentação escolar uma das principais fontes de nutrição para crianças em situação de pobreza. Com a suspensão das aulas, essas crianças tiveram seu acesso à comida de qualidade interrompido, o que pode afetar significativamente seu desenvolvimento cognitivo, em especial nas mais jovens^(19)^, visto que as refeições oferecidas na escola estão positivamente associadas ao bom desempenho acadêmico^(23)^.

Um estudo realizado com estudantes do ensino básico investigou a relação da alimentação gratuita oferecida nas escolas e o desempenho acadêmico. Para isso, os autores fizeram uma análise longitudinal dos resultados de testes que mediram as habilidades em linguagem e matemática. Eles demonstraram que a oferta de alimentação gratuita ou por preço reduzido no período escolar resulta em melhores desempenhos nos testes em questão, tanto para estudantes em situação de pobreza, quanto para os economicamente estáveis^(28)^. Estes dados possibilitam interpretar que, durante a pandemia, estudantes que dependiam da comida oferecida na escola encontram-se em situação de risco quanto ao desenvolvimento das habilidades acadêmicas, além das questões nutricionais e de sobrevivência.

Parte dos artigos também deu atenção aos efeitos de gênero e suas consequências na aprendizagem. As meninas apresentam maior risco de não retornarem à escola devido ao crescimento da taxa de gestação precoce, casamento forçado e exploração sexual. Os autores do estudo reforçam que, durante a epidemia do Ebola, o número de meninas grávidas ao retorno das aulas foi perceptível, das quais muitas foram proibidas de retornar às escolas. Outro aspecto importante é que, durante a pandemia, garotas despendem pelo menos 40% a mais de tempo nos afazeres domésticos, quando comparadas a meninos, o que diminui o tempo dedicado aos estudos e a valorização da educação por parte dos pais^(20)^.

A transição de aulas presenciais para aulas *online* também causou a redução do tempo de instrução, como ainda impossibilitou a participação de alguns estudantes nas atividades propostas pelos professores. A longo prazo, a restrição do tempo de aula pode trazer prejuízos em determinadas habilidades no desempenho em leitura, matemática e ciências^(13)^. Por fim, um aspecto explorado em apenas um artigo foram os efeitos nos estudantes com necessidades educacionais especiais, pois estes se beneficiam significativamente da socialização pelas aulas presenciais^(22)^ e da assistência oferecida, como professores auxiliares e adaptações diárias, o que ainda tem sido desafio aos professores no contexto virtual.

Apesar das contribuições dos artigos já publicados acerca dos prejuízos na aprendizagem de crianças, não foram encontradas pesquisas quanto aos aspectos específicos em relação à aprendizagem escolar da leitura, escrita e matemática. É possível que após o fim da pandemia estas pesquisas sejam realizadas, visto que a volta das crianças à sala de aula possibilitará a verificação dos impactos a longo prazo que a suspensão trouxe na aprendizagem.

Nenhum dos estudos publicados foi produzido no Brasil, ressaltando a necessidade das pesquisas voltarem seus olhares aos aspectos educacionais da pandemia do novo coronavírus, visto que se trata de um país continental com grande desigualdade social.

## CONCLUSÃO

Esta revisão possibilita observar que a suspensão das aulas devido a pandemia do novo coronavírus COVID-19, causará consequências na aprendizagem de crianças com e sem transtornos do neurodesenvolvimento. Não foram encontrados estudos que descrevessem a aprendizagem sob o olhar do desempenho acadêmico da leitura, escrita e matemática, tampouco estudos realizados no Brasil. O foco dos estudos tiveram o olhar dos principais efeitos que irão repercutir na aprendizagem devido às diferenças de acesso entre crianças de alta e baixa renda, por condições desiguais de ensino-aprendizagem e recursos tecnológicos e materiais educacionais para acompanhamento das aulas remotas, bem como realização de estudos no ambiente domiciliar. Outras consequências que impactam a aprendizagem e são descritas como elementos importantes na vida dos estudantes são a falta de alimentação saudável que prejudica o funcionamento cognitivo das crianças, gravidez precoce que impossibilita a continuidade na presença de aulas, exploração sexual de meninas, trabalho infantil e redução do tempo de aula que agravam a influência de questões emocionais na aprendizagem.
